# Measurement error adjusted prevalence of undiagnosed hypertension in England: A cross-sectional study

**DOI:** 10.1371/journal.pone.0359861

**Published:** 2026-10-27

**Authors:** Alejandra Castanon, Andrew Hughes, Katja Grasic, Martin White, James P. Sheppard, Richard J. McManus, Jonathan Pearson-Stuttard, Richard Stevens

**Affiliations:** 1 Health Analytics, Lane Clark & Peacock LLP, London, United Kingdom; 2 Department of Health and Social Care, Office for Health Improvement and Disparities (OHID), London, United Kingdom; 3 Nuffield Department of Primary Care Health Sciences, University of Oxford, Oxford, United Kingdom; 4 Brighton and Sussex Medical School, University of Brighton and University of Sussex, Brighton, United Kingdom; University of Botswana School of Medicine, BOTSWANA

## Abstract

**Background:**

Hypertension case-finding and management is key to reducing premature morbidity and mortality. In England, 14.8% of people receive treatment for hypertension but the total number of hypertensive individuals remains unclear, as it can only be estimated statistically. This study aimed to estimate how many individuals aged 40 + may unknowingly have hypertension by accounting for differences between clinic and out-of-office blood pressure readings.

**Methods:**

We analysed individual-level data from the 2017–2019 Health Survey for England (HSE), including individuals aged ≥40 without a hypertension diagnosis. A validated algorithm (Predicting Out-of-Office Blood Pressure- PROOF-BP) was applied to estimate likely out-of-office measurements. Observed BP was defined as the mean of the second and third measurement taken at an HSE visit. Predicted hypertension was calculated using PROOF-BP and observed hypertension using actual clinic measurements. We explored a diagnostic threshold for predicted (out-of-office) hypertension of ≥135/85 mmHg (primary outcome) and of ≥140/90 mmHg (secondary outcome). Weighted hypertension prevalence estimates are presented by age, sex and geographical region. National population statistics were used to extrapolate results to the English population.

**Results:**

The observed prevalence of undiagnosed hypertension in the study population was 27.7% (95% Confidence Interval [CI]:26.4%−29.1%). The predicted prevalence after adjustment was 21.8% (95%CI:20.6%−23.0%). Predicted prevalence was nearly double in males (29.8%, 95%CI:27.8%−32.0%) compared to females (14.6%, 95%CI:13.3%−15.9%) and varied by region: West Midlands (26.3%, 95%CI:22.2%−31.0%), London (16.9%, 95%CI:13.5%−20.9%). Extrapolating estimates to the total population aged ≥40 in England we estimate approximately 4.2 million people in England are unaware of their hypertension. Of these we estimate that over 2.9 million could be newly diagnosed by the current diagnostic pathway.

**Conclusions:**

Over 4 million people in England are estimated to have undiagnosed hypertension. This study’s refined analytical approach provides a more precise estimate of this population, supporting targeted public health initiatives to identify and address undiagnosed cases.

## Introduction

Hypertension is a major cause of disability and premature death worldwide, [1] increasing the risk of cardiovascular, renal and other diseases [2]. Estimates from the World Health Organisation suggest that up to 46% of adults with hypertension are unaware of their condition [2]. Blood pressure (BP) control significantly reduces cardiovascular disease and associated mortality, [3] highlighting the need for accurate diagnosis and treatment. Hence, hypertension remains one of the most significant preventable causes of morbidity and mortality [4].

Accurately determining the number of individuals unaware of their hypertension is crucial for creating effective strategies to address the condition. However, this is challenging due to its asymptomatic nature and the need to assess “usual blood pressure” over time to reduce misdiagnosis caused by white coat or masked hypertension (also known as measurement error) [5–7]. White coat hypertension occurs when blood pressure readings are elevated in a clinical setting but normal at home, while masked hypertension is the opposite, with normal readings in the clinic but elevated at home, highlighting the importance of consistent monitoring in varied environments. The National Institute for Health and Care Excellence (NICE) guidelines recommend repeated BP measurements for screening, followed by ambulatory blood pressure monitoring (ABPM) or home blood pressure monitoring (HBPM) for confirmation [8].

In England 14.8% of people are currently receiving treatment for hypertension [9], but the total number of hypertensive individuals remains unclear, as it can only be estimated statistically. Current estimates of undiagnosed hypertension in England rely on a single-occasion blood pressure measurements, as collected in the Health Survey for England (HSE) [10]. These estimates do not account for measurement error and since they are based on self-reported treatment may include individuals diagnosed with hypertension but not on medication, leading to potential overestimation of the undiagnosed population [10].

The aim of this study was to estimate the size of the population with undiagnosed hypertension in England, by accounting for potential differences between clinic blood pressure measurements and out-of-office blood pressure measurements. The secondary objective was to estimate the potential number of individuals with undiagnosed hypertension eligible for confirmatory out-of-office blood-pressure measurement under the current NICE diagnostic pathway. Further we explore inequalities in undiagnosed hypertension by age, sex, geography, and deprivation to better guide public health interventions.

## Methods

### Data source

This study used anonymised individual level data from the Health Survey for England (HSE), accessed through the survey year datasets held by the UK Department of Health and Social Care [11–13]. The HSE is an annual representative survey which collects data on a wide range of metrics, through both interviews and physical measurements [14]. Anonymised individual level data from the HSE were accessed via the survey year datasets held by the UK Department of Health and Social Care on 5 June 2024.

For a subset of participants, three blood pressure measurements are taken during a single home visit by a qualified nurse, alongside information on prior hypertension diagnoses and antihypertensive medication use. The HSE employs multi-stage, stratified random probability sampling method, first selecting postcode sectors and then random postal addresses within each sector [14]. To increase sample size and to explore trends over time, data from the 2017, 2018 and 2019 surveys were combined.

The total population in England was taken from the 2021 mid-year population estimates from the Office for National Statistics (ONS) broken down by sex, age and region [15].

This study was a secondary analysis of anonymised HSE data. Ethical approval and informed consent were obtained as part of the original survey. The present study used de-identified data accessed through an approved data-access route; no participants were contacted and no identifiable personal data were accessed.

### Population

The analytical sample was restricted to individuals aged 40 years or older who had three valid blood pressure measurements, had not been diagnosed with hypertension and were not pregnant [14]. Hypertension status was self-reported by survey respondents. Systolic BP measurements above 250 mmHg and below 50 mmHg as well as diastolic BP measurements below 20 mmHg were excluded as deemed implausible. Additional exclusion criteria included those with missing height or weight variables.

### Definitions

Undiagnosed hypertension was defined as hypertension in someone who self-reported no prior hypertension diagnosis [14].

The Predicting Out-of-Office Blood Pressure (PROOF-BP) algorithm [16] predicts likely out-of-office blood pressure measurements based on three BP measurements recorded during a single clinic visit and other patient characteristics (age, sex, weight, height, time since diagnosis, history of cardiovascular disease, hypertension status and antihypertensive medication). The model was designed and validated for use in individuals aged 40 years or older [16]. We defined “predicted” BP (i.e., BP adjusted for measurement error) to be the daytime ambulatory systolic and diastolic blood pressure for each individual predicted by PROOF-BP (using the three valid BP measurements recorded in HSE), and “predicted” hypertension to be predicted BP ≥ 135/85 mmHg. This is the current NICE threshold for diagnosis by measurement in an out-of-office setting [8]. The prevalence of undiagnosed hypertension, among those included in the study, was calculated using predicted BP, and extrapolated to the England population over age 40 years, to meet our primary objective.

For comparison, we also calculate prevalence of undiagnosed hypertension using “observed” blood pressure, which we define to be the mean of the second and third measurement taken at an HSE visit to align with HSE definitions and clinical practice [14]. Since these measurements were conducted by health professionals in a home setting, we applied two thresholds for observed hypertension: ≥ 135/85 mmHg (for consistency with out-of-office-based measures) and ≥140/90 mmHg (a standard clinic threshold) [8].

Our secondary objective was to estimate the extent to which undiagnosed hypertension could be detected through out-of-office monitoring, following current NICE guidelines which combine such screening with an initial office measurement [8]. To examine this, we estimated the prevalence of predicted hypertension cases that also met the observed hypertension clinic threshold (BP ≥ 140/90 mmHg).

Prevalence and absolute numbers were summarised by age, sex and geographical region. Prevalence estimates by deprivation were presented graphically by survey year and age group. Deprivation quintiles were derived from the index of multiple deprivation (IMD) an area-level measure of relative deprivation in England, where quintile 1 denotes the most and quintile 5 the least deprived areas [17]. Due to survey method changes from 2019 onwards, deprivation by age group is presented for 2019 only.

We considered blood pressure to meet the diagnostic threshold for hypertension where either the systolic or diastolic measurements were greater than or equal to the threshold in line with UK Office for National Statistics (ONS) methodology [14].

### Weighting

To account for the complex survey design, the analysis used HSE-provided weights (wt_nurse) to ensure estimates were representative of the target population [15] by sex, age and region, with adjustments for non-response bias. The weights were rescaled to take into account combining data from multiple years of HSE surveys. This approach aligns with the methodology used by ONS to produce current estimates of undiagnosed hypertension [18,19].

### Analyses

Analyses were carried out using R version 4.3.2, employing the ‘survey’ and ‘srvyr’ packages to calculate proportions and adjust the standard errors. Denominators used to calculate prevalence in the study and extrapolations to the population in England are illustrated in Fig 1. To extrapolate results to the English population, all individuals in HSE with a valid BP measurement sample were used as the denominator. The resulting proportion was then multiplied by the total English population over age 40 (n = 28,640,319) [15], taking account of the survey weights. The same methodology was used when estimating the total population in England by sex and age.

**Fig 1:**
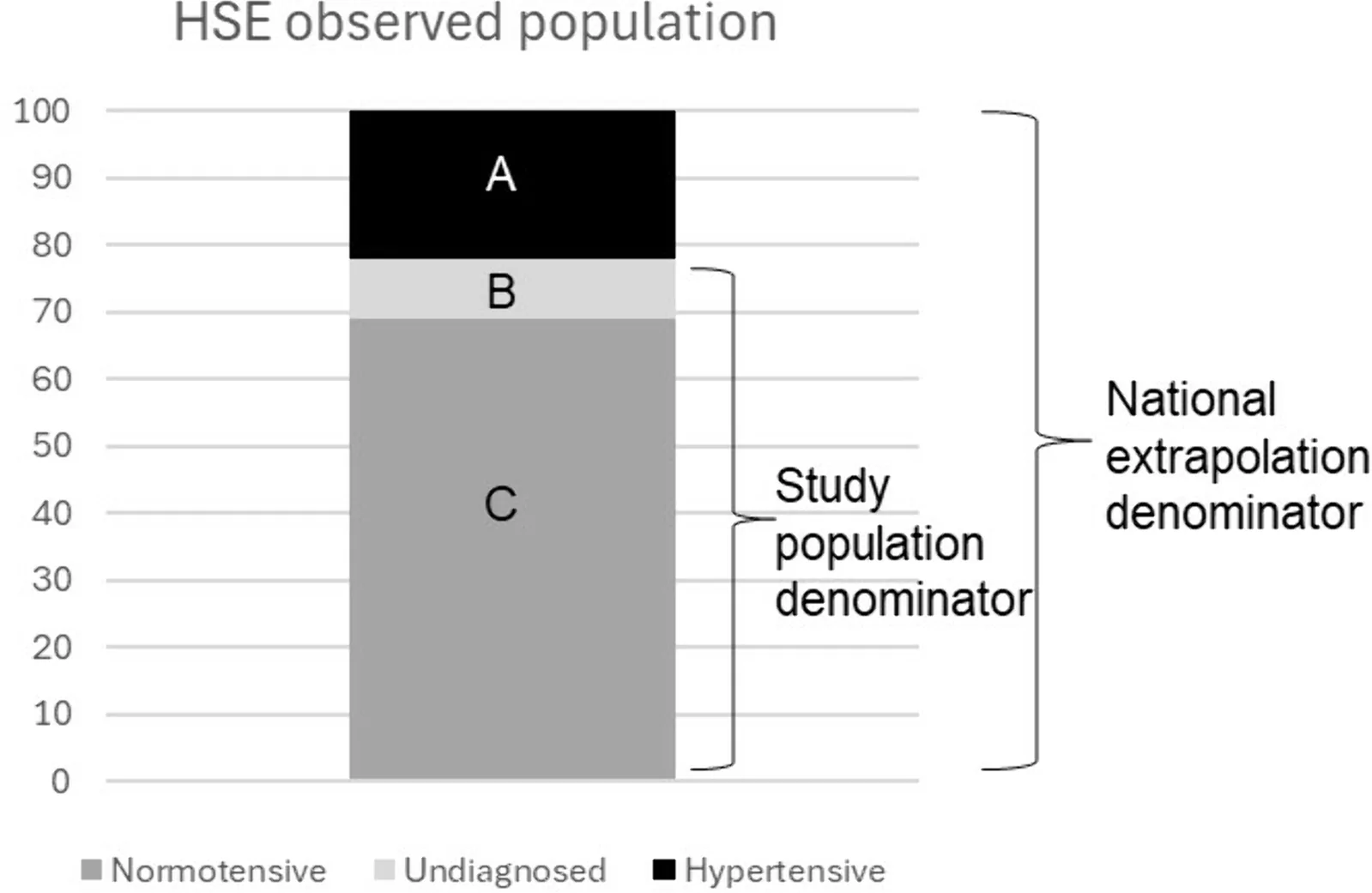
Illustration of study population and denominators. Prevalence in the study (observed and predicted) estimated as B/(B + C). Prevalence in the English population is estimated as B/(A + B + C). The resulting proportion is then multiplied by the total UK population. Note that survey weights were used to account for the complex survey design.

To verify the face validity of the PROOF-BP predictions for blood pressure and hypertension, we compared the overall and age-specific observed and predicted BP distributions across the sample. HSE dataset lacked information on cardiovascular disease (CVD) history, one of the input parameters for determining diastolic BP in the PROOF-BP algorithm. To explore the impact of this, an extreme sensitivity analysis assumed all individuals had CVD and compared it to the primary analysis where all were assumed free of CVD. The analyses is intended as an assessment of the potential influence that lack of CVD history data would have on study outcomes.

## Results

### Description of the unweighted study sample

From a sample of 30,531 individuals in the 2017–2019 HSE, 8,288 were over the aged of 40, with valid blood pressure, height and weight measurements. Among these, 2,821 individuals self-reported a diagnosis of hypertension, resulting in a final analysis sample of 5,467 individuals who self-reported no prior hypertension diagnosis (Fig 2).

**Fig 2:**
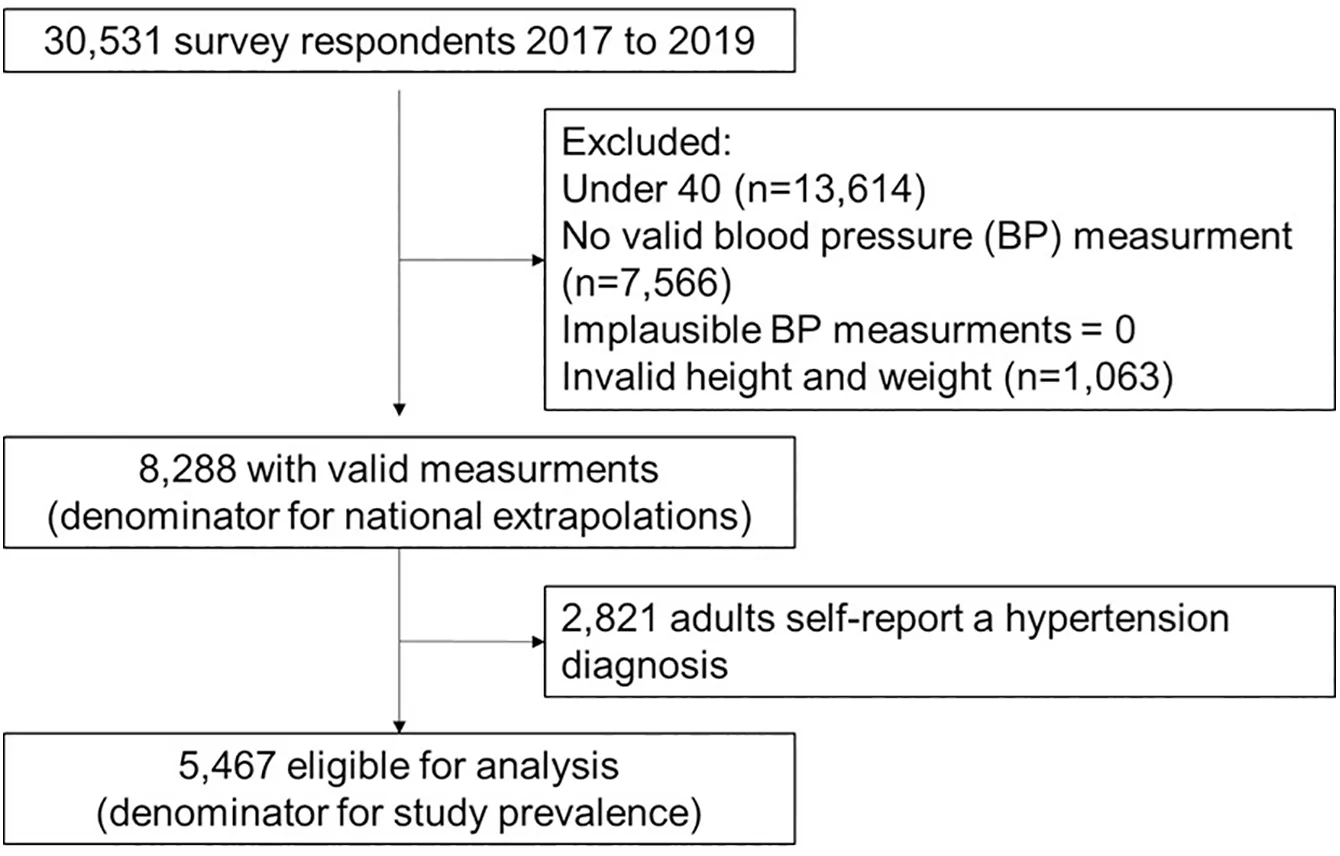
Study inclusion flow chart (unweighted sample).

All subsequent numerical estimates, below, account for survey weighting.

### Prevalence of undiagnosed hypertension (defined as ≥135/85 mm/Hg) among those that self-report no prior hypertension diagnosis

Among individuals who self-reported no prior hypertension diagnosis the observed prevalence of undiagnosed hypertension (without adjustment for measurement error) was 27.7% (95%CI: 26.4% to 29.1%), Table 1.

**Table 1:** Primary analysis - Observed and predicted blood pressures and hypertension (BP ≥ 135/85 mmHg) in survey respondents who self-report no prior hypertension diagnosis, overall and by sex, age group and geographic region.

	Total individuals in sample N	Weighted Sample N	Mean observed BP^1^ mmHg (systolic / diastolic) Mean SD		Percent^2^ of individuals observed^1^ to have a BP ≥ 135/85 mmHg (95% CI)	Mean predicted BP^1^ mmHg (systolic / diastolic) Mean SD		Percent^2^ of individuals predicted^1^ to have a BP ≥ 135/85 mmHg (95% CI)
**Total**	5,467	5,638	125/73	17/12	**27.7 (26.4-29.1)**	128/75	10/7	**21.8 (20.6-23.0)**
**Sex**								
Female	3,140	2,981	123/73	17/11	24.9 (23.3-26.5)	125/73	10/6	14.6 (13.3-15.9)
Male	2,327	2,657	127/74	17/12	30.9 (28.7-33.1)	131/77	9/8	29.8 (27.8-32.0)
**Age group**								
40-44	805	943	119/74	17/13	17.7 (14.6-21.4)	123/77	10/8	11.3 (8.8-14.5)
45-49	781	913	120/74	17/13	20.2 (17.2-23.6)	124/77	10/7	11.0 (8.6-13.9)
50-54	760	918	123/75	17/12	25.6 (22.0-29.6)	126/77	10/7	14.5 (11.6-17.9)
55-59	731	755	125/75	17/12	28.4 (24.7-32.4)	128/76	9/7	19.2 (16.3-22.5)
60-64	644	629	127/74	19/12	31.4 (27.4-35.8)	129/75	10/6	25.8 (22.1-30.0)
65-69	606	506	129/73	17/11	36.2 (32.0-40.6)	131/73	8/6	31.2 (27.2-35.5)
70-74	542	437	131/71	18/10	39.6 (35.0-44.4)	133/72	8/6	38.0 (33.5-42.7)
75-79	304	275	130/68	17/10	37.5 (31.3-44.2)	133/70	8/6	39.0 (32.8-45.6)
80-84	163	143	131/66	19/11	37.3 (29.2-46.3)	136/68	9/7	52.7 (43.7-61.5)
85+	131	119	132/65	20/12	41.7 (32.7-51.3)	138/66	10/8	61.5 (51.9-70.2)
**Region**								
North East	473	277	126/73	13/9	28.5 (24.0-33.4)	128/75	7/5	23.1 (19.3-27.4)
North West	732	739	125/73	20/13	28.0 (24.5-31.8)	128/75	11/8	22.6 (19.3-26.3)
Yorkshire and the Humber	523	533	126/73	16/11	28.9 (24.9-33.1)	128/75	9/7	22.1 (18.4-26.3)
East Midlands	509	485	126/74	15/11	31.8 (27.7-36.1)	129/75	9/7	25.7 (22.2-29.5)
West Midlands	475	546	126/74	20/12	34.5 (29.8-39.6)	129/75	11/7	26.3 (22.2-31.0)
East of England	674	630	125/72	16/11	28.3 (24.9-31.9)	128/74	9/7	23.0 (19.8-26.5)
London	538	771	122/74	19/12	22.2 (18.2-26.8)	126/76	11/8	16.9 (13.5-20.9)
South East	957	1,009	123/72	15/11	25.1 (22.2-28.1)	127/74	9/7	18.7 (16.2-21.5)
South West	586	650	125/73	20/12	27.3 (23.4-31.6)	128/75	11/7	22.6 (18.8-26.9)

^**1**^BP measurements and percentages are weighted, ^**2**^Percentage out of total study sample.

The predicted prevalence of undiagnosed hypertension using the PROOF-BP algorithm to estimate out of office blood pressure was 21.8% (95%CI: 20.6% to 23.0%), Table 1. This predicted prevalence of undiagnosed hypertension varied by age group, with over 50% of eligible individuals aged 80 years and older predicted to be undiagnosed compared to 11% of people in their 40s. Gender and regional differences were notable: the prevalence of undiagnosed hypertension was nearly double in males (29.8%, 95%CI:27.8% to 32.0%) compared to females (14.6%, 95%CI:13.3% to 15.9%) and was higher in the West Midlands (26.3%, 95%CI:22.2% to 31.0%) compared to London (16.9%, 95%CI:13.5% to 20.9%). There was no evidence of a difference in predicted prevalence by deprivation quintile nor calendar year (S1 Fig in S1 File). Similarly, within age groups deprivation quintile did not impact hypertension prevalence (Fig S2 in S1 File).

### Size of the population with undiagnosed hypertension (defined as ≥135/85 mm/Hg) in England

Extrapolating estimates to the total population aged 40 or older, the predicted number of people in England with undiagnosed hypertension was 4,230,495 (Table 2). Although the prevalence of undiagnosed hypertension was greatest in those aged 80 and older in absolute terms the number of individuals with undiagnosed hypertension was smaller in this group (n = 704,133) than in younger age groups; with approximately 1,649,718 individuals aged 40–59, 1,044,234 individuals aged 60–69 and 919,054 aged 70–79 predicted to have undiagnosed hypertension in England.

**Table 2:** Estimation of the prevalence of undiagnosed hypertension in the English population aged 40 and older according to 2021 census data using the combined HSE data for 2017, 2018 and 2019 in the primary and secondary analyses.

	Primary analysis		Secondary analysis			
	**Prevalence of undiagnosed hypertension, using predicted out-of-office BP (≥135/85)**	**Number**^**1**^ **of individuals with undiagnosed hypertension in England, using predicted out-of-office BP (≥135/85)**	**Prevalence of undiagnosed hypertension based on HSE observations (≥140/90)**	**Number**^1^ **of individuals in England with undiagnosed hypertension, using HSE observations (≥140/90)**	**Prevalence of undiagnosed hypertension, based on HSE observations** **(≥140/90) and predicted out-of-office BP (≥135/85)**	**Number**^1^ **of individuals with undiagnosed hypertension in England, using HSE observations (≥140/90) and predicted out-of-office BP (135/85)**
	**% (95%CI)**	**N**	**% (95%CI)**	**N**	**% (95%CI)**	**N**
**Total**	**14.8 (13.9-15.6)**	**4,230,495**	**11.9 (11.1-12.7)**	**3,406,893**	**10.3 (9.5-11.0)**	**2,937,937**
**Age group**						
40-44	10.1 (7.9-13)	364,210	8.5 (6.5-11.2)	306,314	6.7 (4.9-9.0)	240,089
45-49	9.3 (7.3-11.7)	331,142	9.2 (7.2-11.7)	328,616	7.6 (5.8-10.0)	272,490
50-54	11.2 (8.9-13.9)	437,048	10.5 (8.4-13)	408,361	7.8 (6.0-10.1)	305,630
55-59	13.5 (11.5-15.9)	517,318	12.4 (10.4-14.9)	475,577	10.0 (8.1-12.1)	380,356
60-64	16.8 (14.2-19.7)	550,145	13.9 (11.6-16.6)	456,687	12.5 (10.2-15.2)	409,854
65-69	17.8 (15.4-20.4)	494,089	13.9 (11.8-16.3)	386,637	11.9 (10-14.1)	330,345
70-74	19.5 (17.0-22.2)	543,129	15.1 (12.8-17.6)	420,345	14.5 (12.3-17.0)	404,572
75-79	18.0 (14.9-21.7)	375,925	13.1 (10.5-16.2)	272,814	12.4 (9.9-15.4)	258,317
80-84	21.3 (17.1-26.2)	302,741	11.8 (8.7-15.9)	167,842	11.8 (8.7-15.9)	167,842
85+	28.8 (23.4-34.9)	401,392	13.7 (9.9-18.5)	190,639	13.3 (9.6-18.2)	185,941

^**1**^ Among surveyed participants over age 40 according to 2021 census data using the combined HSE data for 2017, 2018 and 2019; note that due to survey weights the totals within the sex and age group categories do not add to the total for all persons 40 + .

### Undiagnosed hypertension detected by out-of-office monitoring carried out according to current NICE guidelines

According to current guidelines, (only) individuals with an initial clinic measurement of ≥140/90 mm/Hg would be offered out-of-office monitoring [8]. At a threshold of ≥140/90 mm/Hg the observed prevalence of undiagnosed hypertension (using survey BP readings) among individuals in this study was 17.5% (95%CI: 16.4% to 18.7%). For these individuals the predicted proportion with hypertension (BP ≥ 135/85 mm/Hg) was 15.1% (95%CI: 14.1% to 16.2%), Table 3. In this secondary analysis the prevalence of undiagnosed hypertension in individuals aged 80–84 years was substantially lower than in the primary analysis (29.2%, 95%CI: 21.8–37.9).

**Table 3:** Secondary analysis estimating percent of individual with hypertension among those who self-report no prior diagnosis, detectable if out-of-office screening was carried out in those with a clinic blood pressure ≥140/90 mmHg, overall and by sex, age group and geographic region.

	Percent of individuals observed^1^ in this study to have a BP ≥ 140/90 mmHg (95% CI)	Percent of individuals observed^1^ in this study to have a BP ≥ 140/90 mmHg and predicted to have APBM of 135/85 mmHg (95% CI)
**Total**	**17.5 (16.4-18.7)**	**15.1 (14.1-16.2)**
**Sex**		
Female	16.0 (14.7-17.4)	12.0 (10.8-13.2)
Male	19.2 (17.5-21.1)	18.7 (16.9-20.5)
**Age group**		
40-44	9.5 (7.2-12.5)	7.5 (5.4-10.1)
45-49	10.9 (8.6-13.9)	9.1 (6.9-11.9)
50-54	13.5 (10.9-16.7)	10.1 (7.8-13.0)
55-59	17.7 (14.7-21.1)	14.1 (11.5-17.2)
60-64	21.4 (17.9-25.5)	19.2 (15.8-23.2)
65-69	24.4 (20.8-28.4)	20.9 (17.5-24.6)
70-74	29.4 (25.2-34.0)	28.3 (24.1-32.9)
75-79	28.3 (22.9-34.5)	26.8 (21.5-32.9)
80-84	29.2 (21.8-37.9)	29.2 (21.8-37.9)
85+	29.2 (21.3-38.6)	28.5 (20.7-37.8)
**Region**		
North East	16.8 (13.5-20.8)	14.2 (11.2-17.8)
North West	18.9 (15.8-22.4)	17.2 (14.3-20.6)
Yorkshire and the Humber	19.4 (16.2-23.2)	16.6 (13.5-20.3)
East Midlands	21.6 (18.0-25.7)	18.4 (15.1-22.3)
West Midlands	20.6 (16.8-25)	17.2 (13.6-21.5)
East of England	16.8 (14.0-20.1)	15.6 (12.9-18.9)
London	12.2 (9.7-15.2)	9.5 (7.2-12.5)
South East	15.6 (13.2-18.3)	13.1 (10.9-15.7)
South West	19.1 (15.8-22.9)	16.9 (13.5-20.9)

^1^BP measurements and percentages are weighted.

Extrapolating estimates to the total population aged 40 or older, these correspond to 3,406,893 million people in England eligible for out-of-office monitoring, of whom 2,937,937 million would be expected to have hypertension (Table 2).

### PROOF-BP model performance

The observed mean systolic BP in the sample was 124.6 mm/Hg (standard deviation (SD) 17.3) and the diastolic BP was 73.2 mm/Hg (SD 11.7). The PROOF-BP model increased the mean systolic BP in the sample by 3.2 mm/Hg and the predicted diastolic BP by 1.8 mm/Hg (S1 Table, S3 and S4 Fig in S1 File). The inclusion of CVD history reduced the predicted mean diastolic BP by 1mgHg (to a mean of 74.0 mm/Hg, SD 6.6), S3 Fig in S1 File. Comparable results were observed by sex and age group (S1 Table in S1 File).

The relationship between BP and age is shown in S5 and S6 Fig in S1 File. The observed and predicted systolic BP follow a linear pattern increasing over time, with predicted values higher than observed values. The observed diastolic mean BP increases till age 50–54 and decreases thereafter. However, the predicted diastolic BP changes this pattern showing decreasing diastolic BP with age. Assuming the entire population has a history of CVD made predicted BP values for those under age 60 closer to the observed but substantially increased BP values above observed for older individuals.

## Discussion

After adjusting for measurement error, we estimate that over four million adults aged 40 or older in England have hypertension but do not know it (defined as an out-of-office measurement ≥135/85 mm/Hg). Significant differences in the prevalence of undiagnosed hypertension were found across sex, age and geography.

NICE guidelines recommend out of office measurement to confirm the diagnosis of hypertension (at a cut-off of ≥135/85 mmHg) among those that have clinic BP between 140/90 mmHg and 180/120 mmHg [8]. Under current guidelines, we estimated that 2.9 million people in England could be newly diagnosed by the current diagnostic pathway. A further 1.3 million individuals would have an out-of-office measurement ≥135/85 mmHg but be below the clinic threshold where they would be recommended by a clinician for an out-of-office confirmation of a diagnosis.

The analytical approach in this study enhances accuracy and reliability in estimating the undiagnosed population size. By accounting for both white coat and masked hypertension, it provides a clearer view of true hypertension prevalence. Out-of-office measurements are also better correlated with cardiovascular outcomes than clinic-based readings, further strengthening the relevance of these findings to public health strategy [20,21].

### Research in context

Estimates in this study, while focused on those aged 40 and older and thus not directly comparable to published estimates, align with broader HSE-based findings, including regional disparities in undiagnosed hypertension and a greater likelihood of undiagnosed hypertension in males.[18,22]

Our primary analysis corresponds to an estimate of the distribution of blood pressures across the whole study population; the secondary analysis studies only those that would be observed under current guidelines. Therefore, the primary analysis principally handles ‘masked’ hypertension (i.e., spuriously low blood pressure measured in clinic but raised at home), whilst the secondary analysis addresses the ‘white coat’ hypertension (i.e., spuriously high blood pressure measured in clinic but normal at home) [23,24].

Undiagnosed hypertension was estimated to be particularly prevalent among individuals aged 80 or older, a group with good evidence for treatment [25]. The estimated effect of adjustment for measurement error was particularly large for those aged 80–84 (extra 23.5% with hypertension) and those aged 80+ (extra 33.0% with hypertension; Table 1 columns 7 vs. 5). However, the impact would be mitigated by NICE guidance that a higher treatment threshold (clinic BP ≥ 150/90 mmHg) be used in individuals aged 80 years or older. At younger ages the lack of evidence on which to determine whether treatment should be indicated for all individuals with clinic BP measurements of between 135/85 mmHg and ≤140/90 mmHg is reflected in the fact that they would not be treated under current guidance [8]. Notwithstanding all individuals with BP ≥ 135/85 mmHg will benefit from lifestyle advice to avoid a future hypertension diagnosis.

The mean BP predicted by PROOF-BP was on average slightly higher than the observed BP (Table 1, row 1). This finding, counter to intuition, is probably partly because we are studying a selected population – those without known hypertension – but also because the true distribution is more complex than captured by means alone (S1 Fig in S1 File). Although the phenomenon of higher blood pressure in clinic (the “white coat effect”) is better known than the converse, true differences between clinic and out-of-clinic blood pressures can vary greatly. A systematic review found that, across different studies in different settings, differences in blood pressure could vary from −5 to +25 mmHg systolic and from −3 to +10 mmHg diastolic [26]. Nevertheless in this study predicted BP values resulted in lower prevalence of undiagnosed hypertension, suggesting that estimates of undiagnosed hypertension based on research measurements may overestimate the true prevalence.

### Policy implications

The finding that an estimated 1.6 million individuals aged 40–59 have undiagnosed hypertension is concerning, especially given data suggesting delays in diagnosis lead to increased cardiovascular events [27,28]. Previous reports suggest that individuals least likely to have hypertension—such as younger adults, those in good general health, and those who were not overweight or obese—were the most likely to remain undiagnosed if they did have hypertension [18].

The Health Survey for England is instrumental in shaping public health policy and tracking health trends within the English population. Accurate estimates of the size of the population who are unaware that they have hypertension are important to enable targeted public health action. We have shown that after adjusting for potential measurement error there are still millions of individuals with hypertension that do not know it. Better estimates of the population size impact can assist decision-makers in making evidence-based decisions about the need for new or revised blood pressure screening programs and in conducting cost-effectiveness analyses.

### Strengths and limitations

Our predicted estimates, derived from a peer-reviewed and validated model (PROOF-BP), [16] provide measurement-adjusted blood pressure predictions, enhancing accuracy. This methodology can be easily incorporated into routine statistics, allowing policymakers to access precise, replicable statistics on undiagnosed hypertension. Notably, our study estimated the size of the entire population with undiagnosed hypertension, beyond those identified under current diagnostic protocols. The PROOF-BP model validity has been tested among individuals aged 40 and older, its performance cannot be assumed to be equivalent in younger populations. This consideration informed the decision to exclude those aged under 40 years from the study.

We were unable to report results by ethnicity because these data were under reported in HSE. Although previous research is not conclusive, there is some indication that undiagnosed hypertension might differ by ethnicity. Published literature suggests the white coat effect might be lower in some South Asian populations such as Bangladeshis but report no differences between white and black populations [29]. Other literature using HSE found no difference in prevalence of undiagnosed hypertension by ethnicity [22].

PROOF-BP was derived for use with measurements by health professionals in an office setting. HSE measurements were taken in a household setting but conducted under research conditions by health professionals. There is no accepted diagnostic threshold for hypertension when measurements are taken this way. Our analysis shows that however the data are treated, there are likely to be millions of adults aged 40 and older with undiagnosed hypertension in England.

PROOF-BP was externally validated in its intended clinical context, its application to HSE data is subject to uncertainty which is further compounded by the lack of data on CVD history. S3 Fig in S1 File suggests that the lack of information on this predictor had limited impact on model performance in the HSE context. The absence of CVD data would be expected to result in under-classification of hypertension, primarily among individuals where diastolic BP is elevated but systolic BP remains within normal limits. However, in older adults where CVD history is more prevalent, isolated systolic hypertension becomes increasingly common, largely reflecting age-related stiffening of the large arteries, which is associated with rising systolic blood pressure and stable or declining diastolic blood pressure.

It should be noted that the sensitivity analysis is intentionally implausible and is not applicable to direct ambulatory blood-pressure measurements nor should they be interpreted as fully validated estimates for the HSE population.

## Conclusion

This study used a validated tool to adjust national undiagnosed population statistics for blood pressure measurement error, to provide reliable estimates of the size of the problem and an indication of where inequalities in diagnoses exist. The methodology described in this study is easily adaptable and implementable facilitating the generation of evidence to inform policy and ensure early diagnosis and appropriate control of hypertension avoiding costly and life-threatening adverse cardiovascular outcomes.

## Supporting information

S1 File Supplemental Tables and Figures. (DOCX)
