# Supplementary material for: Measurement error adjusted prevalence of undiagnosed hypertension in England: A cross-sectional study
Source: PLoS One. 2026 Oct 27;21(10):e0359861. doi: 10.1371/journal.pone.0359861 (PMC13712611; doi:10.1371/journal.pone.0359861)
Supplement: Supplementary file 1 [file pone.0359861.s001.docx]

Supplemental Tables and Figures

For manuscript titled “Measurement error adjusted prevalence of undiagnosed hypertension in England: a cross-sectional study”.

Contents

[Table S1. Mean (and standard deviation) of blood pressure measurements for survey respondents with self-reported undiagnosed hypertension, mean of observed values and predicted values, overall and by sex and age group.	2](#_Toc239062544)

[Figure S1. Proportion with hypertension (95% CI) by deprivation and HSE calendar year among individuals using predicted out-of-office blood pressure ≥135/85.	4](#_Toc239062545)

[Figure S2. Proportion with hypertension (95% CI) by deprivation and age group among individuals using predicted out-of-office blood pressure ≥135/85 (based in 2019 IMD scores)	5](#_Toc239062546)

[Figure S3. Density distribution of diastolic blood pressure including observed, predicted assuming no participants have a history of cardiovascular disease (CVD) and predicted assuming all participants has a history of CVD.	6](#_Toc239062547)

[Figure S4. Density distribution of observed and predicted systolic blood pressure.	7](#_Toc239062548)

[Figure S5: Diastolic blood pressure mean values by age group.	8](#_Toc239062549)

[Figure S6: Systolic blood pressure mean values by age group.	9](#_Toc239062550)

### Table S1. Mean (and standard deviation) of blood pressure measurements for survey respondents with self-reported undiagnosed hypertension, mean of observed values and predicted values, overall and by sex and age group.

|  | **Weighted N** | **Observed systolic** | | **Predicted systolic** | | **Observed diastolic** | | **Predicted diastolic** | | **Predicted diastolic (all CVD)** | |
| --- | --- | --- | --- | --- | --- | --- | --- | --- | --- | --- | --- |
|  |  | **Mean** | **SD** | **Mean** | **SD** | **Mean** | **SD** | **Mean** | **SD** | **Mean** | **SD** |
| **Total** | 5638 | 124.6 | 17.3 | 127.7 | 10.1 | 73.2 | 11.7 | 75.0 | 7.3 | 74.0 | 6.6 |
| **Sex** |  |  |  |  |  |  |  |  |  |  |  |
| Female | 2981 | 122.6 | 16.8 | 124.9 | 9.6 | 72.6 | 10.7 | 73.3 | 6.0 | 72.4 | 5.7 |
| Male | 2657 | 126.8 | 16.5 | 130.8 | 9.2 | 73.9 | 12.2 | 76.8 | 7.5 | 75.7 | 6.8 |
| **Age groups** |  |  |  |  |  |  |  |  |  |  |  |
| 40-44 | 943 | 119.0 | 16.7 | 122.9 | 10.4 | 73.6 | 13.3 | 76.9 | 7.8 | 73.2 | 7.8 |
| 45-49 | 913 | 120.4 | 17.3 | 124.3 | 10.2 | 74.3 | 12.9 | 76.6 | 6.8 | 73.8 | 6.8 |
| 50-54 | 918 | 122.8 | 17.4 | 126.2 | 9.9 | 74.8 | 12.4 | 76.6 | 7.2 | 74.7 | 7.2 |
| 55-59 | 755 | 125.2 | 16.7 | 127.8 | 8.9 | 74.5 | 12.1 | 75.8 | 6.7 | 74.8 | 6.7 |
| 60-64 | 629 | 127.0 | 18.8 | 129.5 | 9.7 | 74.1 | 11.6 | 75.2 | 6.3 | 75.1 | 6.3 |
| 65-69 | 506 | 129.1 | 17.2 | 130.9 | 8.3 | 72.5 | 10.8 | 73.4 | 6.1 | 74.2 | 6.1 |
| 70-74 | 437 | 131.5 | 17.9 | 133.1 | 8.1 | 71.2 | 10.3 | 72.0 | 5.9 | 73.7 | 5.9 |
| 75-79 | 275 | 130.1 | 16.7 | 133.4 | 8.0 | 68.4 | 10.3 | 69.6 | 6.0 | 72.1 | 6.0 |
| 80-84 | 143 | 131.0 | 19.3 | 135.8 | 9.3 | 66.3 | 11.5 | 68.1 | 7.3 | 71.5 | 7.3 |
| 85+ | 119 | 131.9 | 19.7 | 138.0 | 10.1 | 65.3 | 11.6 | 66.1 | 7.5 | 70.6 | 7.5 |
| **Region** |  |  |  |  |  |  |  |  |  |  |  |
| North East | 277 | 125.6 | 13.1 | 128.3 | 7.3 | 72.7 | 9.3 | 74.6 | 5.4 | 73.5 | 5.0 |
| North West | 739 | 125.1 | 20.1 | 128.1 | 11.4 | 73.1 | 12.6 | 74.9 | 7.9 | 73.9 | 7.1 |
| Yorkshire and the Humber | 533 | 125.9 | 15.7 | 128.2 | 9.3 | 73.4 | 11.4 | 75.0 | 7.2 | 74.0 | 6.4 |
| East Midlands | 485 | 126.2 | 14.7 | 128.9 | 9.0 | 73.9 | 11.4 | 75.2 | 7.0 | 74.5 | 6.3 |
| West Midlands | 546 | 126.3 | 19.6 | 128.6 | 11.2 | 74.4 | 12.3 | 75.5 | 7.4 | 74.4 | 6.8 |
| East of England | 630 | 124.8 | 15.6 | 128.1 | 8.6 | 72.3 | 11.1 | 74.3 | 6.8 | 73.6 | 5.5 |
| London | 771 | 121.6 | 18.5 | 126.0 | 11.1 | 73.8 | 12.5 | 75.9 | 8.0 | 74.3 | 7.5 |
| South East | 1009 | 123.4 | 15.1 | 126.9 | 9.2 | 72.3 | 11.2 | 74.4 | 7.0 | 73.4 | 6.2 |
| South West | 650 | 124.9 | 19.5 | 128.0 | 11.5 | 73.3 | 11.8 | 74.9 | 7.2 | 74.2 | 6.8 |

### Figure S1. Proportion with hypertension (95% CI) by deprivation and HSE calendar year among individuals using predicted out-of-office blood pressure ≥135/85.

### Figure S2. Proportion with hypertension (95% CI) by deprivation and age group among individuals using predicted out-of-office blood pressure ≥135/85 (based in 2019 IMD scores)

### Figure S3. Density distribution of diastolic blood pressure including observed, predicted assuming no participants have a history of cardiovascular disease (CVD) and predicted assuming all participants has a history of CVD.

### Figure S4. Density distribution of observed and predicted systolic blood pressure.

### Figure S5: Diastolic blood pressure mean values by age group.

### Figure S6: Systolic blood pressure mean values by age group.
