# Supplementary material for: Measurement error adjusted prevalence of undiagnosed hypertension in England: A cross-sectional study
Source: PLoS One. 2026 Oct 27;21(10):e0359861. doi: 10.1371/journal.pone.0359861 (PMC13712611; doi:10.1371/journal.pone.0359861)
Supplement: Supplementary file 2 [file pone.0359861.s002.docx]

Dr. Alejandra Castanon and colleagues analysed individual-level data from the 2017 to 2019 Health Survey for England (HSE). The major finding was that an estimated over 4 million people in England had undiagnosed hypertension.

1. Please confirm and revise the incorrect value of 69.4% on line 45.
2. The PROOF-BP algorithm relies on three sequential BP measurements, yet this study defines observed BP as the average of only the second and third readings. This methodological mismatch may drive disparities in results and should be clearly described in the manuscript.
3. Please provide a clear definition and explanation of the deprivation quintile classification adopted in this study.
4. Please adjust the manuscript content to meet the formatting requirements of the journal.
