# Supplementary material for: Measurement error adjusted prevalence of undiagnosed hypertension in England: A cross-sectional study
Source: PLoS One. 2026 Oct 27;21(10):e0359861. doi: 10.1371/journal.pone.0359861 (PMC13712611; doi:10.1371/journal.pone.0359861)
Supplement: Supplementary file 3 [file pone.0359861.s004.docx]

*Measurement error adjusted prevalence of undiagnosed hypertension in England: a cross-sectional study*

Dear Editor,

We are grateful to the reviewers for taking the time to read and comment on our manuscript and for the opportunity to respond to their comments. A point-by-point response can be found bellow with the accompanying changes tracked in the revised manuscript.

| **Number** | **Reviewer** | **Comment** | **Response** |
| --- | --- | --- | --- |
| 1 | Reviewer 1 | The authors used a validated prediction model (PROOF-BP) to estimate out-of-office BP. This prediction model needs cardiovascular history as one of the information inputed into it. However, the HSE survey results (secondary data) which the authors used, did not collect cardiovascular history. The authors say they did sensitivity analysis to account for that. However, the sensitivity analysis does not justify using a prediction model with important missing data. It makes the results invalid. | We thank the reviewer for highlighting this important limitation. The PROOF-BP model includes cardiovascular disease (CVD) history as a predictor; however, a directly comparable CVD history variable was not available in the HSE datasets used for this analysis. Our primary analysis therefore necessarily relied on an assumption regarding this model input.  We agree that the sensitivity analysis does not fully resolve the absence of this predictor and have revised the manuscript to make this clearer. Specifically, we have removed language which could imply that the sensitivity analysis validates the primary estimate. Instead, we now present this analysis as an extreme-scenario assessment of the potential influence of the unavailable CVD history variable.  However, we disagree that it makes the results invalid. In this analysis, assigning a history of CVD to all participants resulted in a modest reduction in mean predicted diastolic blood pressure (BP) compared to the predicted BP without considering CVD history, from 75.0mmHg (SD 7.3) to 74.0 mmHg (SD 6.6 mmHg). We have clarified that this scenario is intentionally implausible and should not be interpreted as an estimate of the true prevalence of cardiovascular disease in the study population.  We have added a more prominent limitation in the Discussion: as follows “PROOF-BP was externally validated in its intended clinical context, its application to HSE data is subject to uncertainty which is further compounded by the lack of data on CVD history. S3 Fig suggests that the lack of information on this predictor had limited impact on model performance in the HSE context. The absence of CVD data would be expected to result in under-classification of hypertension, primarily among individuals where diastolic BP is elevated but systolic BP remains within normal limits. However, in older adults where CVD history is more prevalent, isolated systolic hypertension becomes increasingly common, largely reflecting age-related stiffening of the large arteries, which is associated with rising systolic blood pressure and stable or declining diastolic blood pressure.  It should be noted that the sensitivity analysis is intentionally implausible and is not applicable to direct ambulatory blood-pressure measurements nor should they be interpreted as fully validated estimates for the HSE population.” |
| 2 | Reviewer 1 | It will be helpful to the reader if all the objectives of the study are stated in one section of the write up. The aim of the study is stated in line 80. Then line 129 talks about secondary objective, but there is no mention what the primary objective is. | We agree. The objectives were previously described in separate parts of the manuscript, which may have obscured the distinction between the primary and secondary objectives. We have revised the final paragraph of the Introduction to state all objectives together.  The last paragraph of the introduction now reads:  “The aim of this study was to estimate the size of the population with undiagnosed hypertension in England, by accounting for potential differences between clinic blood pressure measurements and out-of-office blood pressure measurements. **The secondary objective was to estimate the potential number of individuals with undiagnosed hypertension eligible for confirmatory out-of-office blood-pressure measurement under the current NICE diagnostic pathway.** Further we explore inequalities in undiagnosed hypertension by age, sex, geography, and deprivation to better guide public health interventions.” |
| 3 | Reviewer 1 | Line 77-79 seem to imply that all those treated for hypertension should be having controlled BP. Hypertension statistics globally show that many people have uncontrolled hypertension despite treatment. | We thank the reviewer. We agree that the distinction between diagnosed hypertension, antihypertensive treatment and blood-pressure control requires clearer wording. Our intention was not to imply that people with diagnosed hypertension, including those receiving treatment, necessarily have controlled blood pressure. Rather, the study focused on people without a reported hypertension diagnosis.  We have revised the paragraph as follows:  “Current estimates of undiagnosed hypertension in England rely on a single-occasion blood pressure measurements, as collected in the Health Survey for England (HSE).10 These estimates do not account for measurement error and since they are based on self-reported treatment may include individuals diagnosed with hypertension but not on medication, leading to potential overestimation of the undiagnosed population.10” |
| 4 | Reviewer 1 | Line 285 has been written as a blanket statement, but it is only true for those people 75 years or older. | Line 285 in the submitted article reads “The mean BP predicted by PROOF-BP was on average slightly higher than the observed BP (Table 1, row 1). This finding, counter to intuition, is probably partly because we are studying a selected population – those without known hypertension – but also because the true distribution is more complex than captured by means alone (Figure S1).  The predicted higher BP is observed across all age groups, both in Table 1 and Table S1.  We have corrected an error in referencing supplemental figures at the end of the paragraph. Originally it references Figure S1 but should reference Figures S3 and S4. |
| 5 | Reviewer 1 | Line 319-321: "Because causes of hypertension in younger populations tend to be secondary to other conditions, PROOF-BP is unlikely to accurately predict blood pressure among those aged under 40 years...". This is not true for people of African ethnicity, who studies have shown, have more primary rather than secondary hypertension across all age groups. | We thank the reviewer and agree that the original statement was overly broad. The study does not examine the aetiology of hypertension, and it is not appropriate to infer that hypertension in younger adults is generally secondary to another condition. We have therefore removed this statement.  Instead, the discussion now reads  “The PROOF-BP model validity has been tested among individuals aged 40 and older, its performance cannot be assumed to be equivalent in younger populations. This consideration informed the decision to exclude those aged under 40 years from the study.” |
| 6 | Reviewer 1 | Line 336: "Further, diastolic BP plays a smaller role in diagnosing hypertension, particularly in older individuals where CVD history is more prevalent". This is not true because diastolic BP determines pulse pressure which is a key determinant of cardiovascular risk. Diastolic BP is an indicator of the stiffness of central arteries in relation to peripheral arteries, hence the tendency for older individuals to have isolated systolic hypertension (where DBP is lower than 80 mmHg) as a result of increased stiffening of the large central arteries. Therefore the authors downplayed the importance of diastolic BP (line 334-335). | We thank the reviewer and agree that the wording could be interpreted as downplaying the clinical and prognostic importance of diastolic blood pressure. We have revised the relevant text.  We have removed the statement that diastolic BP “plays a smaller role in diagnosing hypertension” and replaced it with the following:  “S3 Fig suggests that the lack of information on this predictor had limited impact on model performance in the HSE context. The absence of CVD data would be expected to result in under-classification of hypertension, primarily among individuals where diastolic BP is elevated but systolic BP remains within normal limits. However, in older adults where CVD history is more prevalent, isolated systolic hypertension becomes increasingly common, largely reflecting age-related stiffening of the large arteries, which is associated with rising systolic blood pressure and stable or declining diastolic blood pressure.” |
| 7 | Reviewer 2 | 1.Please confirm and revise the incorrect value of 69.4% on line 45. | We thank the reviewer for spotting this error. We have removed the percentage from the results in the abstract to align with the results presented within the manuscript.  The sentence now reads:  “Of these we estimate that over 2.9 million could be newly diagnosed by the current diagnostic pathway.” |
| 8 | Reviewer 2 | 2.The PROOF-BP algorithm relies on three sequential BP measurements, yet this study defines observed BP as the average of only the second and third readings. This methodological mismatch may drive disparities in results and should be clearly described in the manuscript. | We appreciate that the methods did not fully describe our approach. In the primary analyses we used PROOF-BP to predict BP based on three BP measurements recorded in HSE. The text has been updated as follows:  “We defined “predicted” BP (i.e. BP adjusted for measurement error) to be the daytime ambulatory systolic and diastolic blood pressure for each individual predicted by PROOF-BP (**using the three valid BP measurements recorded in HSE**), and “predicted” hypertension to be predicted BP ≥135/85 mmHg.  It is only when calculating the “observed BP” that we use the second and third measurements because this aligns with HSE definitions and what happens in clinical practice. For clarity the text has been amended as follows:  “For comparison, we also calculate prevalence of undiagnosed hypertension using “observed” blood pressure, which we define to be the mean of the second and third measurement taken at an HSE visit **to align with HSE definitions and clinical practice**.^14^ |
| 9 | Reviewer 2 | 3.Please provide a clear definition and explanation of the deprivation quintile classification adopted in this study. | We thank the reviewer for highlighting that we have not provided information on the deprivation index used. The following text has been added:  “Deprivation quintiles were derived from the index of multiple deprivation (IMD) an area-level measure of relative deprivation in England, where quintile 1 denotes the most and quintile 5 the least deprived areas.”  We have added the following reference:  [English indices of deprivation 2019 - GOV.UK](https://www.gov.uk/government/statistics/english-indices-of-deprivation-2019) |
| 10 | Reviewer 2 | 4.Please adjust the manuscript content to meet the formatting requirements of the journal. | This has been implemented. |
| 11 | Editor | . Please ensure that your manuscript meets PLOS ONE's style requirements, including those for file naming. The PLOS ONE style templates can be found at <https://journals.plos.org/plosone/s/file?id=wjVg/PLOSOne_formatting_sample_main_body.pdf> and [https://journals.plos.org/plosone/s/file?id=ba62/PLOSOne_formatting_sample_title_authors_affiliations.pdf](https://eur01.safelinks.protection.outlook.com/?url=https%3A%2F%2Ftrack.editorialmanager.com%2FCL0%2Fhttps%3A%252F%252Fjournals.plos.org%252Fplosone%252Fs%252Ffile%253Fid%3Dba62%252FPLOSOne_formatting_sample_title_authors_affiliations.pdf%2F1%2F010f019ef7b0c1af-926ba9da-c7dc-47ac-8452-26e3f9059576-000000%2Filom9KYwLw3GaqsQCy3ey3wJFvYEzvc7VLyNontFb-k%3D258&data=05%7C02%7Calex.castanon%40lcp.uk.com%7C326c96db80bb482e4baa08ded1a16114%7C77c0b9a377fd44d787b62b6621d23e83%7C0%7C0%7C639178688509729256%7CUnknown%7CTWFpbGZsb3d8eyJFbXB0eU1hcGkiOnRydWUsIlYiOiIwLjAuMDAwMCIsIlAiOiJXaW4zMiIsIkFOIjoiTWFpbCIsIldUIjoyfQ%3D%3D%7C0%7C%7C%7C&sdata=Sj5dfXRabBUqLTL%2Fr9IUcggn0UmakLJEvhFpLpBrw%2BM%3D&reserved=0) | We thank the reviewer. The manuscript has been reformatted to comply with PLOS ONE requirements, including title-page structure, section headings, reference style, figure and table presentation, supporting-information citations and file naming. We have also reviewed the manuscript against the PLOS ONE formatting templates specified by the Editor. |
| 12 | Editor | 2. Please note that PLOS One has specific guidelines on code sharing for submissions in which author-generated code underpins the findings in the manuscript. In these cases, we expect all author-generated code to be made available without restrictions upon publication of the work. Please review our guidelines at <https://journals.plos.org/plosone/s/materials-and-software-sharing#loc-sharing-code> and ensure that your code is shared in a way that follows best practice and facilitates reproducibility and reuse. | We confirm that author-generated analytic code underpinning the findings will be made openly available without restriction upon publication. The code will be deposited in GitHub, with documentation sufficient to reproduce the analysis for authorised users of the underlying HSE data.  The HSE data are third-party data accessed through the UK Data Service and are subject to its access and licensing conditions; therefore, the individual-level data cannot be redistributed by the authors. This restriction does not apply to the author-generated code.  There are some internal compliance steps to go through to create the GitHub repository. While they are underway we did not want to delay submitting the response to reviewers. We will add the link at proof stage.  In the manuscript we have added to the Data and code availability section the following:  “Author-generated analytic code underpinning the findings is available here: Placeholder [link to be added at proof stage]” |
| 13 | Editor | 3. [Thank you for stating in your Funding Statement: “This research was funded in part, by the Wellcome Trust [211182/Z/18/Z] awarded to James Sheppard. The funders played no role in the study design, data collection and analysis, decision to publish, or preparation of the manuscript.”  Please provide an amended statement that declares *all* the funding or sources of support (whether external or internal to your organization) received during this study, as detailed online in our guide for authors at http://journals.plos.org/plosone/s/submit-now. Please also include the statement “There was no additional external funding received for this study.” in your updated Funding Statement. Please include your amended Funding Statement within your cover letter. We will change the online submission form on your behalf.](Thank%20you%20for%20stating%20in%20your%20Funding%20Statement:%20%0b) | Addressed it now reads:  “This research was funded in part, by the Wellcome Trust [211182/Z/18/Z] awarded to J Sheppard. The funders played no role in the study design, data collection and analysis, decision to publish, or preparation of the manuscript.  LCP provided support in the form of salaries for Jonathan Pearson-Stuttard, Katia Grasic and Alejandra Castanon**, but did not have any additional role in the study design, data collection and analysis, decision to publish, or preparation of the manuscript. The specific roles of these authors are articulated in the ‘author contributions’ section.**  There was no additional external funding received for this study |
| 14 | Editor | 4. Thank you for stating the following in the Competing Interests section: “During this study, James P Sheppard received funding from the Wellcome Trust/Royal Society via a Sir Henry Dale Fellowship (ref: 211182/Z/18/Z), the National Institute for Health and Care Research (NIHR) and from the British Heart Foundation (refs: PG/21/10341; FS/19/13/34235).  Richard Stevens receives funding from the National Institute for Health and Care Research (NIHR) Applied Research Collaboration Oxford and Thames Valley at Oxford Health NHS Foundation Trust.  Jonathan Pearson-Stuttard is Partner and Head of Health Analytics at Lane Clark & Peacock LLP, Chair of the Royal Society for Public Health. Alejandra Castanon is an employee of Lane Clark & Peacock LLP.  Richard J McManus is an NIHR Senior Investigator. | Acknowledged |
| 15 | Editor | For the purpose of open access, the author has applied a CC BY public copyright license to any Author Accepted Manuscript version arising from this submission. The views expressed are those of the author(s) and not necessarily those of the NHS, NIHR or the Department of Health and Social Care.”  We note that one or more of the authors are employed by a commercial company:  Lane Clark & Peacock LLP  a. Please provide an amended Funding Statement declaring this commercial affiliation, as well as a statement regarding the Role of Funders in your study. If the funding organization did not play a role in the study design, data collection and analysis, decision to publish, or preparation of the manuscript and only provided financial support in the form of authors' salaries and/or research materials, please review your statements relating to the author contributions, and ensure you have specifically and accurately indicated the role(s) that these authors had in your study. You can update author roles in the Author Contributions section of the online submission form.  Please also include the following statement within your amended Funding Statement. “The funder provided support in the form of salaries for authors [insert relevant initials], but did not have any additional role in the study design, data collection and analysis, decision to publish, or preparation of the manuscript. The specific roles of these authors are articulated in the ‘author contributions’ section.” If your commercial affiliation did play a role in your study, please state and explain this role within your updated Funding Statement.  b. Please also provide an updated Competing Interests Statement declaring this commercial affiliation along with any other relevant declarations relating to employment, consultancy, patents, products in development, or marketed products, etc.  Within your Competing Interests Statement, please confirm that this commercial affiliation does not alter your adherence to all PLOS ONE policies on sharing data and materials by including the following statement: "This does not alter our adherence to PLOS ONE policies on sharing data and materials.” (as detailed online in our guide for authors <http://journals.plos.org/plosone/s/competing-interests>) . If this adherence statement is not accurate and there are restrictions on sharing of data and/or materials, please state these. Please note that we cannot proceed with consideration of your article until this information has been declared.  Please include both an updated Funding Statement and Competing Interests Statement in your cover letter. We will change the online submission form on your behalf. | Addressed, see response to point 13 |
|  | Editor | 5. Please note that your Data Availability Statement is currently missing the DOI/accession number of each dataset or a direct link to access each database. If your manuscript is accepted for publication, you will be asked to provide these details on a very short timeline. We therefore suggest that you provide this information now, though we will not hold up the peer review process if you are unable. | The data availability statement has been updated as follows:  The data underlying the results presented in the study are available from the UK Data Service.  NatCen Social Research, University College London, Department of Epidemiology and Public Health. (2023). *Health Survey for England*. [data series]. *7th Release.* UK Data Service. SN: 2000021, [http://doi.org/10.5255/UKDA-SN-8488-3](https://eur01.safelinks.protection.outlook.com/?url=http%3A%2F%2Fdoi.org%2F10.5255%2FUKDA-SN-8488-3&data=05%7C02%7CAlex.Castanon%40lcp.com%7C0799bee57e5842a0874508deffa24c5a%7C77c0b9a377fd44d787b62b6621d23e83%7C0%7C0%7C639229271341103325%7CUnknown%7CTWFpbGZsb3d8eyJFbXB0eU1hcGkiOnRydWUsIlYiOiIwLjAuMDAwMCIsIlAiOiJXaW4zMiIsIkFOIjoiTWFpbCIsIldUIjoyfQ%3D%3D%7C0%7C%7C%7C&sdata=6g9oe278RG7R%2FOVgm1gPm680hSs%2B3lAHCE8hZH7Il9Y%3D&reserved=0)  Datasets used in this study include:  Health Survey for England, 2017 -  [DOI: http://doi.org/10.5255/UKDA-SN-8860-1](http://doi.org/10.5255/UKDA-SN-8860-1)  Health Survey for England, 2018 - [DOI: http://doi.org/10.5255/UKDA-SN-8649-2](http://doi.org/10.5255/UKDA-SN-8649-2)  Health Survey for England, 2019 - [DOI: http://doi.org/10.5255/UKDA-SN-8860-1](http://doi.org/10.5255/UKDA-SN-8860-1) |
|  | Editor | 6. Please include your full ethics statement in the ‘Methods’ section of your manuscript file. In your statement, please include the full name of the IRB or ethics committee who approved or waived your study, as well as whether or not you obtained informed written or verbal consent. If consent was waived for your study, please include this information in your statement as well. | We have added the following ethics statement to the study methods:  This study was a secondary analysis of anonymised HSE data. Ethical approval and informed consent were obtained as part of the original survey. The present study used de-identified data accessed through an approved data-access route; no participants were contacted and no identifiable personal data were accessed. |
|  | Editor | 7. [Please include captions for your Supporting Information files at the end of your manuscript, and update any in-text citations to match accordingly. Please see our Supporting Information guidelines for more information: http://journals.plos.org/plosone/s/supporting-information.](Please%20include%20captions%20for%20your%20Supporting%20Information%20files%20at%20the%20end%20of%20your%20manuscript,%20and%20update%20any%20in-text%20citations%20to%20match%20accordingly.%20Please%20see%20our%20Supporting%20Information%20guidelines%20for%20more%20information:%20http:/journals.plos.org/plosone/s/supporting-information.%20) | Included at the end of the manuscript |
|  | Editor | 8. If the reviewer comments include a recommendation to cite specific previously published works, please review and evaluate these publications to determine whether they are relevant and should be cited. There is no requirement to cite these works unless the editor has indicated otherwise. | Not applicable |
